# Supplementary material for: Integrated photonic metasystem for image classifications at telecommunication wavelength
Source: Nat Commun. 2022 Apr 19;13:2131. doi: 10.1038/s41467-022-29856-7 (PMC9018697; doi:10.1038/s41467-022-29856-7)
Supplement: Supplementary file 1 — Supplementary Information [file 41467_2022_29856_MOESM1_ESM.pdf]

Supplementary Information for

Integrated photonic metasystem for image classifications at telecommunication wavelength

Zi Wang, Lorry Chang, Feifan Wang, Tiantian Li and Tingyi Gu*
Department of Electrical and Computer Engineering, University of Delaware, Newark, DE 19711, USA
*Email: tingyigu@udel.edu

Supplementary Note 1: Integrated photonic frameworks for matrix operation
Supplementary Note 2: PyTorch-based training of the diffractive optical network
Supplementary Note 3: Broadband operation of the integrated spatial pattern classifier
Supplementary Note 4: Metasystem scalability and accuracy

Supplementary Note 1: Integrated photonic frameworks for matrix operation

Vector-by-matrix multiplication (VMM) is one of the fundamental operations in the accelerator hardware [1]. Supplementary Table 1 compares the VMM power efficiency, throughput, and footprint of integrated photonic circuits based on Mach-Zehnder interferometer (MZI), microring resonators (MRRs), and metasystem (this work).

Supplementary Table 1: Integrated photonic frameworks for VMM.

Method for matrix operation	Multi-wavelength modulation and summation [2-4]	Singular value decomposition [5]	Diffraction equation (This work)
Device architecture	MRR weight banks [2, 4] and directional couplers [3]	MZI[5]	Cascaded metasurfaces
Signal processing	Optoelectronic	Optoelectronic	All-optical
Weight matrix	16×16 [3]	4×4 [5]	450×2
Footprint (mm^2)	16 [3]	0.75[5]	0.135
Throughput (Tb/s)	11 [2]	1 [5]	5
Insertion loss	27dB [3]	Not reported	15 dB (average)
Operational power	17fJ per MAC	1pJ per FLOP	10^{-5} fJ per FLOP

Calculations of throughout and power consumption in Supplementary Table 1 are detailed below.

Throughput: The equation (S1) described the optical data path limited operation frequency:

$$f_{DPATH} = \frac{1}{\tau} = \frac{1}{S/v} \qquad (S-1)$$

Where τ is the latency, S is the distance of the optical data path, v is the speed of light propagation in the media. Throughput of the system is calculated by:

$$Throughput = n_i f_{DPATH} \qquad (S-2)$$

where n_i is the dimension of the input data. After such calculation, the system could reach a throughput of $5 * 10^{12} b/s$. The number of operations per second of the system is:

$$FLOPS = mNNf_{DPATH}, \qquad (S-3)$$

where N is the neuron number per layer, and m is the layer number [6].

Operational power: The power consumption of the metasystem is the summation of the power required for propagation and the optical power required to support an optical nonlinearity that could be potential implementations of future devices. If we assume a saturable absorber threshold of $p \cong 1MW/cm^2$ (e.g. graphene) and an area of a neuron A = $1\mu m^2$, the total power needed for nonlinearity is estimated to be $P = p \times A \times N = N(mW)$. For the proposed two-layer system, the power consumption is:

$$\frac{P + Loss}{FLOPS} = \frac{450 \times 1.9 \times 10^{-3}}{2 \times 450 \times 450 \times 5 \times 10^{12}} \cong 4.2 \times 10^{-19} J\ per\ FLOP. \qquad (S-4)$$

Where *Loss* is the insertion loss of the system.

Supplementary Table 2: Comparison of neuron networks-based image classifiers

Neuron network	Convolution Neural Network [7]		Convolution Neural Network [8]	Diffraction Neural network (This work)
Programmed layer(s)	One amplitude-only layer of DMD		One phase-only layer of diffractive optic elements	Phase-only layers of metasurface
Reconfigurable	Yes		No	No
Postprocessing	Required		Required	Maximal only
Hyperspectral	No		Possible	Yes
Kernel size	16×208×208		16×32×32	450×2
Dataset	MNIST	CIFAR	CIFAR-10	MNIST
Accuracy	98%(s)	63%(s)	51% (e)	96% (s)

(s): numerical simulation result. (e): experimental measurements.

Supplementary Note 2: PyTorch-based training of the diffractive optical network

2.1 Design framework

The metasystem is designed by the PyTorch framework (Facebook, Inc.) [9]. A spatial pattern classifier is used to illustrate the design process (Supplementary Figure 1a). Additional random phase noise was added in the input array and each hidden layer to represent the fabrication variation (orange blocks in Supplementary Figure 1) and the coupling related phase fluctuations (light yellow block in Supplementary Figure 1). The metasystem was trained to be robust against experimental variations [10].

In each pixel of the letter image, the input information was encoded as the amplitude of the input electromagnetic (EM) field with their default phase values set at zero. Examples of the training and testing data of the inputs are illustrated in Supplementary Figure 1b. As shown in Supplementary Figure 1a, the input electric fields are multiplied with the complex-valued modulation generated by random phase noise (uniform distribution on the interval $[0, 0.5\pi)$, to be discussed in section S2.5) and the desired phase shift of each layer, and after the 2D free-space propagation, the output field of each layer is fed to the next layer as the input field. For the last layer, we calculated the cross-entropy loss between the intensity of the output field and the ground truth of the input data.

We used the Adam algorithm [11] in back-propagation and update the neurons of the network to minimize the loss function (Supplementary Figure 1c). The whole forward/ backward propagation process can be calculated by matrix multiplication. The electric field of the layer $(l+1)$ can be calculated by

$$[m^{l+1}(1),\ m^{l+1}(2), \dots,\ m^{l+1}(p),\ \dots,\ m^{l+1}(n)] = [t^l(1)\cdot m^l(1),\ \dots, t^l(i)\cdot m^l(i), \dots, t^l(k)\cdot m^l(k)] \cdot \begin{bmatrix} w^l(1,1) & \cdots & w^l(1,n) \\ \vdots & \ddots & \vdots \\ w^l(k,1) & \cdots & w^l(k,n) \end{bmatrix}, \qquad (S-5)$$

where $m^l(p)$ is the input electric field of the p-th neuron in the layer l, $t^l(p) = a \cdot \exp(j\phi^l(p))$ is the complex transmission coefficient of the p-th neuron in the layer l, and $w^l(p,q)$ is the propagation coefficient from the p-th neuron in layer l to the q-th neuron in the layer $(l+1)$ which can be derived from Eqs. (2) in the main text, as shown in Supplementary Figure 1c.

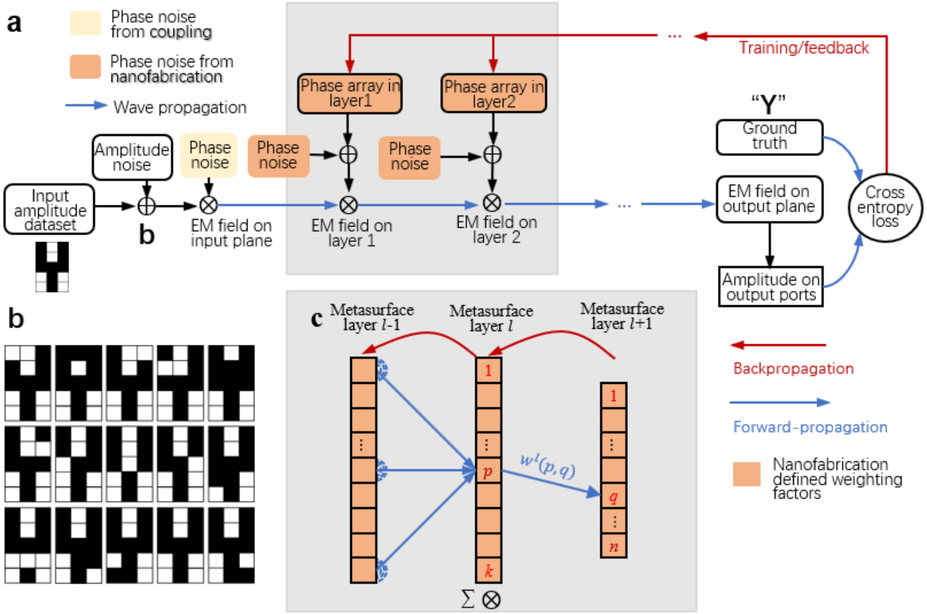

Supplementary Figure 1. The framework of designing a metasystem classifier robust against fabrication variations. (a) The complex electromagnetic (EM) field propagates freely in the x-y plane. The complex EM field of each layer's output is multiplied with the phase modulation by the metasurface layer (orange) and random phase noise (blue). The multiplied signal is propagated and transferred to the next layer (blue arrows). (b) Example training and testing input data of "Y" with different amplitude noise**.** (c) Details of the in-plane free propagation module (grey shaded area in a). The inter-layer connectivity is described by diffraction equations.

2.2 2D-1D matrix compression

As shown in Eqs. (2) in the main text, the propagation coefficient $w^l(p,q)$ is the function of the interlayer distance and the distance along y direction. Because the layers in our system are in the same length and parallel to each other, the matrix W became a Toeplitz matrix [12], as shown in the following equation:

$$W = \begin{bmatrix} w^l(1,\ 1) & \cdots & w^l(1,\ n) \\ \vdots & \ddots & \vdots \\ w^l(n,1) & \cdots & w^l(n,\ n) \end{bmatrix} = \begin{bmatrix} w_0 & w_1 & \cdots & w_{n-1} \\ w_1 & w_0 & & \vdots \\ \vdots & & \ddots & w_1 \\ w_{n-1} & \cdots & w_1 & w_0 \end{bmatrix}. \qquad (S-6)$$

Here we create a circulant matrix $C(w')$ using a vector w' which is composed of the elements in the matrix W as follows:

$$w' = [w_0,\ w_1,\ \ldots,\ w_{n-1},\ 0,\ w_{n-1},\ \ldots,\ w_2,\ w_1]^T \qquad (S-7)$$

and

$$C(w') = \begin{bmatrix} w_0 & w_1 & \cdots & w_{n-2} & w_{n-1} & & \\ w_1 & w_0 & w_1 & & w_{n-2} & & \\ \vdots & w_1 & w_0 & \ddots & \vdots & \cdots & * \\ w_{n-2} & & \ddots & \ddots & w_1 & & \\ w_{n-1} & w_{n-2} & \cdots & w_1 & w_0 & & \\ & & \vdots & & & & \\ & & * & & & W & \end{bmatrix} = \begin{bmatrix} W & * \\ * & W \end{bmatrix}, \qquad (S-8)$$

where matrix W is a Toeplitz matrix as shown in Eqs. (S-6). We need to calculate the multiplication of the matrix W and an $(n \times 1)$ vector v as shown in Eqs. (S-5). Here we show a fast way to do the calculation by calculating $C(w') \cdot v'$

$$C(w') \cdot v' = \begin{bmatrix} W & * \\ * & W \end{bmatrix} \cdot \begin{bmatrix} v \\ 0 \end{bmatrix} = \begin{bmatrix} W \cdot v \\ * \cdot v \end{bmatrix} \qquad (S-9)$$

and get the first n rows of the result which is $W \cdot v$. We can calculate $C(w') \cdot v'$ by following:

$$C(w') \cdot v' = \mathrm{ifft}\big(\mathrm{fft}(w') .\cdot \mathrm{fft}(v')\big), \qquad (S-10)$$

where *fft* and *ifft* represent Fourier and inverse Fourier transforms, respectively. w' is the $(2n \times 1)$ vector in Eqs. (S-7), and v' is a $(2n \times 1)$ vector composed with the vector v and a zero vector with the dimension of $(n \times 1)$. Instead of using the matrix W with the dimension of $(n \times n)$, we used the matrix w' with the dimension of $(2n \times 1)$ which reduced the requirement for both calculation and data storage. As the propagation matrix W is the most computationally expensive component, the proposed system reduces the number of metasurface cells from $O(N^2)$ to $O(N)$.

2.3 Metasurface cell design

The transmission and the phase shift of the transmitted light can be modified by changing the geometric parameters of the slots as shown in Supplementary Figure 2. Also, Supplementary Figure 3 shows that the phase shift is not sensitive to the incident angle. Phase deviation less than 0.02×2π is found at the incident angle of 40°.

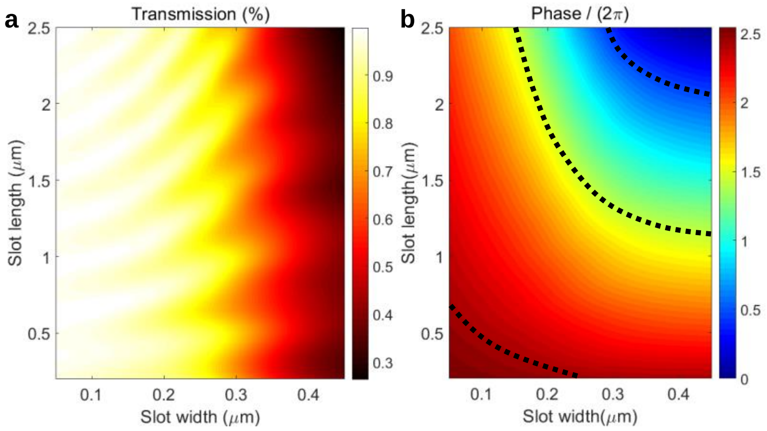

Supplementary Figure 2. Fully programmable complex transmission coefficient of the metasurface cell. The simulated (a) transmission and (b) phase shift versus slot length and width. Dashed curves share the same phase, and spaced by 2π.

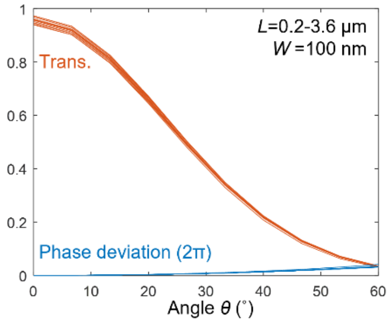

Supplementary Figure 3. Incident angle-dependent transmission and phase deviation of the slots with a fixed width of 100 nm and varying lengths (0.2-3.6μm). The result is obtained by 3D FDTD simulation.

2.4 Loss function and optimizer definition

The softmax of the output intensity is defined as:

$$S_i = \frac{e^{a_i}}{\sum_k e^{a_k}}, \qquad (S-9)$$

and the cross-entropy loss is defined as

$$L = -\sum_i y_i * \log(S_i), \qquad (S-8)$$

where $GT = [y_1, y_2, \dots, y_i, \dots]$ is the ground truth of the input data, and $a_i = |m^M(i)|^2$ is the output intensity. The gradient can be derived as

$$\frac{\partial L}{\partial(|m^{M+1}|^2)} = S - GT, \qquad (S-9)$$

and

$$\frac{\partial(|m^{M+1}|^2)}{\partial\phi} = 2\cdot real\left\{m^{M+1}\cdot\frac{\partial\left(m^{M+1^*}\right)}{\partial\phi}\right\}, \qquad (S-10)$$

where ϕ is the phase shifts of the neurons, m^{M+1^*} is the complex conjugate of m^{M+1}.

2.5 Interval range for phase noise robustness

The interval incorporates the fabrication offsets. During the ebeam lithography and etching process, random variations of slot width and length <10 nm are expected, and more variation can be expected for inter-layer distance (100μm ± 10 nm). Besides the error caused by fabrication, the experiment setup and measurement can also cause the error. A larger interval provides better robustness against error but also decreases the classification accuracy. The interval [0,0.5π) is selected for balancing the design robustness and accuracy.

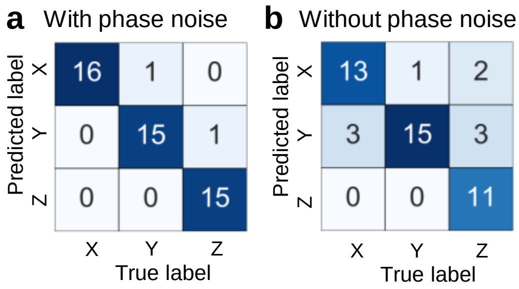

Supplementary Figure 4. Comparison of classification accuracy with and without considering the phase noise. (a) and (b) The FDTD simulated confusion matrices for pattern classification systems with and without the phase noise added during the training step.

Supplementary Note 3: Broadband operation of the integrated spatial pattern classifier

We verified the broadband property of the classifier neural network in both simulations and experiments as shown in Supplementary Figure 5. Even though the classifier neural network is designed around 1550nm, it can also work with the input wavelength of 1520nm and 1620nm. The overall output intensity for the input wavelength at 1500nm and 1600nm is lower than the input wavelength at 1550nm.

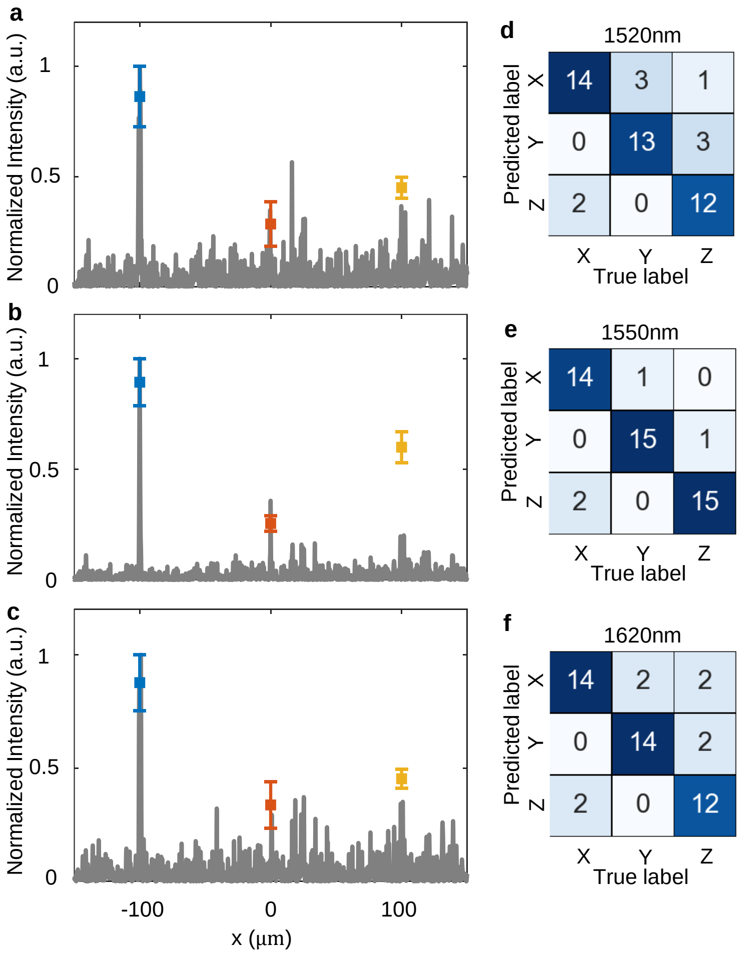

Supplementary Figure 5. Broadband operation of the classifier. FDTD simulated spatial distribution of light on the output plane (grey lines) compared with experimental results (squares with error bar), as the wavelength of the input c.w. laser set at (a) 1520 nm, (b) 1550 nm and (c) 1620 nm. The error bars represent the standard deviation (s.d.) for 16 measurements. The experimentally measured values are marked as blue, red, and yellow dots for the output port representing 'X', 'Y', 'Z' respectively. The corresponding confusion matrixes of the experiments are shown in (d), (e), and (f).

Supplementary Note 4: Metasystem scalability and accuracy

The scalability of the design algorithm is verified by a more complicated system of a Modified National Institute of Standards and Technology (MNIST) handwritten digit database. The accuracy of the output is evaluated by Python simulations. Supplementary Figure 6 compares the loss functions and truth tables of the small scale (15-pixel inputs for 'XYZ') and large scale (784-pixel inputs for MNIST). The proposed system in section S1 is efficient. The

accuracy of the small-scale system converges after 1 Epoch and only 3 epoch brings the accuracy of a larger system to be 96%. Compared with the free-space diffractive neural network [13-14], our structure has fewer neurons and a faster training speed.

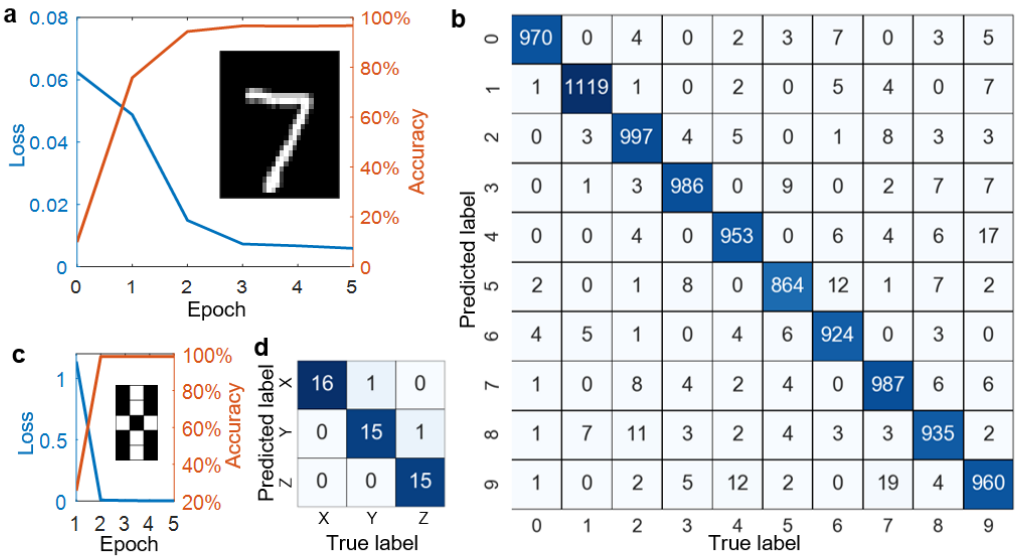

Supplementary Figure 6. Letters and handwriting digits (MNIST) recognition training for phase-only ID²NN. (a) Training convergence plots. Inset: example of input and (b) confusion matrix for MNIST handwriting digits with 784 pixels, and (c-d) letters images with 15 pixels.

At a fixed number of total weight elements, the system accuracy dramatically improves with the depth of the system (the numbers of the diffraction layers) [15]. We investigated the system accuracy's sensitivity to the number of layers, and the interlayer spacing (Supplementary Figure 7). A metasystem with 5 layers and 4000 neurons per layer achieves an accuracy of more than 96%. With the same number of neurons, the metasystem accuracy significantly improves in a multi-layer system (Supplementary Figure 7a). The interlayer spacing (D) increases the connectivity, and thus the system accuracy (Supplementary Figure 7b). At least 1000 neurons per layer and 3 layers are needed for maintaining high accuracy of 92%. A trade-off between the device accuracy and the metasystem footprint is observed.

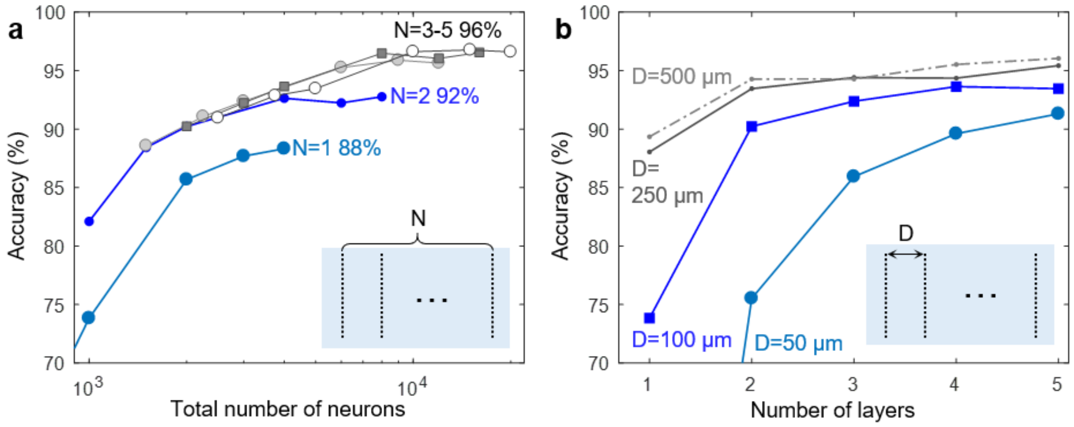

Supplementary Figure 7. Scalability of the integrated diffractive optical network for MNIST. (a) Simulated accuracy versus the same total number of the weight elements, with a different number of layers (N). The inter-layer distance is 100 μm. **(b)** Accuracy versus layer number with inter-layer distance (D) of 50, 100, 250, and 500 μm. The number of phase shifters per layer is 1000.

Supplementary References

[1] Kitayama, K. et al. Novel frontier of photonics for data processing—Photonic accelerator. APL Photonics 4, 090901 (2019).

[2] Xu, X. et al. 11 TOPS photonic convolutional accelerator for optical neural networks. Nature 589, 44–51 (2021).

[3] Feldmann, J. et al. Parallel convolutional processing using an integrated photonic tensor core. Nature 589, 52–58 (2021).

[4] Tait, A. N. et al. Neuromorphic photonic networks using silicon photonic weight banks. Sci. Rep. 7, 7430 (2017).

[5] Shen, Y. et al. Deep learning with coherent nanophotonic circuits. Nat. Photonics 11, 441–446 (2017).

[6] Goi, E. et al. Nanoprinted high-neuron-density optical linear perceptrons performing near-infrared inference on a CMOS chip. Light Sci Appl 10, 40 (2021).

[7] Miscuglio, M. et al. Massively parallel amplitude-only Fourier neural network. Optica 7, 1812 (2020).

[8] Chang, J., Sitzmann, V., Dun, X., Heidrich, W. & Wetzstein, G. Hybrid optical-electronic convolutional neural networks with optimized diffractive optics for image classification. Sci Rep 8, 12324 (2018).

[9] Paszke, A. et al. PyTorch: An Imperative Style, High-Performance Deep Learning Library. arXiv:1912.01703 (2019).

[10] Pai, S. et al. Parallel Programming of an Arbitrary Feedforward Photonic Network. IEEE J. Select. Topics Quantum Electron. 26, 1–13 (2020).

[11] Kingma, D. P. & Ba, J. Adam: A Method for Stochastic Optimization. arXiv:1412.6980 (2017).

[12] Golub, G. H. & Van Loan, C. F. Matrix computations. (The Johns Hopkins University Press, 2013).

[13] Backer, A. S. Computational inverse design for cascaded systems of metasurface optics. Opt. Express 27, 30308 (2019).

[14] Zhu, D., Liu, Z., Raju, L., Kim, A. S. & Cai, W. Multifunctional Meta-Optic Systems: Inversely Designed with Artificial Intelligence. arXiv:2007.00130 [physics] (2020).

[15] Mengu, D., Luo, Y., Rivenson, Y. and Ozcan, A., Analysis of diffractive optical neural networks and their integration with electronic neural networks, IEEE J. Sel. Top. Quant. 26, 1-14 (2019).
